# Overall and Cause-Specific Mortality in Patients With Type 1 Diabetes Mellitus: A Population-Based Cohort Study in Taiwan From 1998 Through 2014

**DOI:** 10.2188/jea.JE20200026

**Published:** 2021-09-05

**Authors:** Chin-Li Lu, Ya-Hui Chang, Santi Martini, Ming-Fong Chang, Chung-Yi Li

**Affiliations:** 1 Graduate Institute of Food Safety, College of Agriculture and Natural Resources, National Chung Hsing University, Taichung, Taiwan; 2 Department of Public Health, College of Medicine, National Cheng Kung University, Tainan, Taiwan; 3 Department of Epidemiology, Faculty of Public Health, Universitas Airlangga, Surabaya, Indonesia; 4 Department of Family Medicine, Tainan Sin-Lau Hospital, Tainan, Taiwan; 5 Department of Public Health, College of Public Health, China Medical University, Taichung, Taiwan; 6 Department of Healthcare Administration, College of Medical and Health Science, Asia University, Taichung, Taiwan

**Keywords:** cohort studies, mortality, standardized mortality ratio, type 1 diabetes, underlying cause of death

## Abstract

**Background:**

To investigate all-cause and cause-specific mortality in Taiwanese patients with type 1 diabetes.

**Methods:**

A cohort of 17,203 patients with type 1 diabetes were identified from Taiwan’s National Health Insurance claims in the period of 1998–2014. Person-years were accumulated for each individual from date of type 1 diabetes registration to date of death or the last day of 2014. Age, sex, and calendar year standardized mortality ratios (SMRs) were calculated with reference to the general population.

**Results:**

In up to 17 years of follow-up, 4,916 patients died from 182,523 person-years. Diabetes (30.15%), cancer (20.48%), circulatory diseases (13.14%), and renal diseases (11.45%) were the leading underlying causes of death. Mortality rate (26.93 per 1,000 person-years) from type 1 diabetes in Taiwan was high, the cause of death with the highest mortality rate was diabetes (8.12 per 1,000 person-years), followed by cancer (5.52 per 1,000 person-years), and circulatory diseases (3.54 per 1,000 person-years). The all-cause SMR was significantly elevated at 4.16 (95% confidence interval, 4.04–4.28), with a greater all-cause SMR noted in females than in males (4.62 vs 3.79). The cause-specific SMR was highly elevated for diabetes (SMR, 16.45), followed by renal disease (SMR, 14.48), chronic hepatitis and liver cirrhosis (SMR, 4.91) and infection (SMR, 4.59). All-cause SMRs were also significantly increased for all ages, with the greatest figure noted for 15–24 years (SMR, 8.46).

**Conclusions:**

Type 1 diabetes in both genders and all ages was associated with significantly elevated SMRs for all-cause and mostly for diabetes per se and renal disease.

## INTRODUCTION

Type 1 diabetes mellitus is associated with a high risk of premature death from various acute and chronic causes.^1^^–^^3^ Causes of death in children and young adults with type 1 diabetes are mainly related to acute diabetic complications; meanwhile, the main cause of death in adulthood is related to long-term complications, particularly cardiovascular disease (CVD).^4^^,^^5^ Although the risk of mortality in individuals with type 1 diabetes remains elevated, a declined trend in mortality of type 1 diabetes population was noted in many parts of the world, such as Norway, Australia, and Sweden.^6^^–^^8^ A meta-analysis of the relative risk (RR) of mortality for type 1 diabetes compared with the general population that included 26 studies with 88 sub-populations found that the overall RR of mortality was 3.82 (95% confidence interval [CI], 3.41–4.29) compared with the general population.^9^ Observations using data prior to 1971 had a considerably larger estimated RR (5.80) when compared with the data between 1971 and 1980 (RR 5.06), 1981–1990 (RR 3.59), and those after 1990 (RR 3.11). The recently improved mortality from type 1 diabetes is primarily due to enforced guidelines that emphasize tight glycemic control, blood pressure control, and treatment of dyslipidemia, as well as smoking cessation, in the management of type 1 diabetes.^10^^–^^12^

Recent evidence suggests a reduction in mortality from chronic complications,^8^ but little change in mortality from acute complications of type 1 diabetes.^13^ Mortality varies noticeably among countries,^4^ and countries with a lower incidence of type 1 diabetes have higher absolute and relative mortality than higher incidence countries.^6^^,^^14^ Willi et al^15^ suggested ethnic disparities in the outcomes of children with type 1 diabetes, with black participants having more diabetic ketoacidosis and severe hypoglycemic events than white or Hispanic participants. A recent systematic review identified 16 studies that showed racial/ethnic minority youth with type 1 diabetes had higher hemoglobin A1c (HbA1c) than Caucasian youth.^16^ A recent review indicated that South Asian ethnicity with type 1 diabetes have higher mortality than white Europeans due to excess CVD. Type 1 diabetes in South Asian also have significantly higher HbA1c, lower high-density lipoprotein, and lower rates of neuropathy than white Europeans.^17^

In Taiwan, the annual incidence rate of childhood (<15 years) type 1 diabetes was stable for boys and girls, with a mean annual incidence rate of 5.3 per 100,000 children between 2003 and 2008.^18^^,^^19^ Compared with Western countries, especially the Nordic nations, Taiwan is among the nations with low incidence rate of type 1 diabetes. However, mortality of individuals with type 1 diabetes in Taiwan has not been adequately studied. This study aimed to investigate the overall, sex-specific, and age-specific risks of mortality from all-cause and various causes among type 1 diabetes patients in 1998–2014.

## METHODS

The study proposal was approved by the Institutional Review Board of National Cheng Kung University Hospital (No. B-EX-105-010). A written informed consent was waived because of de-identification of personal identity.

### Data sources

Data analyzed in this study were retrieved from datasets of the National Health Insurance (NHI) program and Taiwan Death Registry (TDR) in the period of 1998–2014. The NHI claim datasets store the inpatient/outpatient medical claims of all residents of Taiwan, and the NHI Administration performs quarterly expert reviews on a random sample of medical claims to ensure their accuracy.^20^

We used several parts of the NHI claim datasets, including the Catastrophic Illness Database (CID) and the Beneficiary Registry that included sociodemographic characteristics of every individual. Information of type 1 diabetes diagnosis is among the listed catastrophic illnesses in the CID. Individuals who are registered in the CID for type 1 diabetes must report to the NHIA review board a physician’s diagnosis certificate and relevant medical records, including examination results, fasting or glucagon-stimulated C-peptide level, anti-GAD antibody level, and history of diabetic ketoacidosis. Type 1 diabetes diagnosis in the CID was previously used to report the incidence of type 1 diabetes in Taiwan.^18^^,^^19^ with a positive predictive rate of 98.3%.^19^

In Taiwan, all live births and deaths should be registered within 10 days after birth or death as a legal requirement. Death certificates include various information, including demographic variables, underlying cause of death (UCOD), place of death, and marital status. Data quality for TDR have been evaluated and are considered valid and complete.^21^

### Study design

We used a retrospective cohort study design that initially included 17,269 individuals with type 1 diabetes registered with CID between 1998 and 2014. After excluding 66 patients with missing information on gender or age at CID registration, this study enrolled 17,203 study subjects. Date on CID registration was regarded as date of cohort enrollment (ie, cohort entry).

The study patients were linked to the TDR using unique personal identification number for identifying the patients who died during the study period of 1998–2014. The information on the UCOD was based on the International Classification of Diseases, Ninth Revision Clinical Modification (ICD-9-CM) (1998–2007) or the Tenth Revision (ICD-10-CM) (2008–2014) codes. In the 17 years of observation, 4,916 individuals died, including 2,511 males and 2,405 females.

### Statistical analysis

The person-years observed for each study subject were accumulated from date of cohort enrollment to date of death or the last day of 2014. Age at enrollment was categorized into 0–14, 15–29, 30–44, and ≥45 years. The person-years were then categorized according to calendar year, gender, and patient’s age at different calendar years (ie, prior to 2003, 2003–2006, 2007–2010, and 2011–2014). The study cohort contributed 182,523 person-years during the follow-up period. Mortality rate was calculated as the number of death divided by observed person-years. Survival curves for sex-stratified and age-stratified cumulative survival risk were plotted using the Kaplan-Meier product-limit method and compared using log-rank test.

We compared patients’ risks of all-cause and cause-specific mortality to those of the general population with comparable sex and age at specific calendar years. The UCOD analyzed in this study included diabetes, circulatory diseases, malignant neoplasm, renal diseases, violence and accidents, suicide, infection, chronic hepatitis or liver cirrhosis, and chronic obstructive pulmonary disease (COPD). eTable 1 shows the codes for the selected UCOD analyzed in this study.

The expected number of death for the type 1 diabetes cohort was calculated from the person-year approach using the age group and sex-specific annual mortality rates, with reference to the general population of Taiwan. The annual age- and sex-specific population sizes for the general population during the study period were derived from the national annual household registration statistics published by Ministry of the Interior of Taiwan. The annual average size of the general population during the study period (ie, 1998–2014) was 23,769,198. Overall, sex-specific, and age-specific standardized mortality ratios (SMRs) were calculated. The 95% CI for SMR was estimated using the exact estimation.^22^ The distributions of UCOD were graphically presented according to age at enrollment, gender, and year of cohort entry. The analysis was performed using SAS (version 9.4; SAS Institute, Cary, NC, USA), and level of significance was set at α = 0.05.

## RESULTS

**Figure 1.**
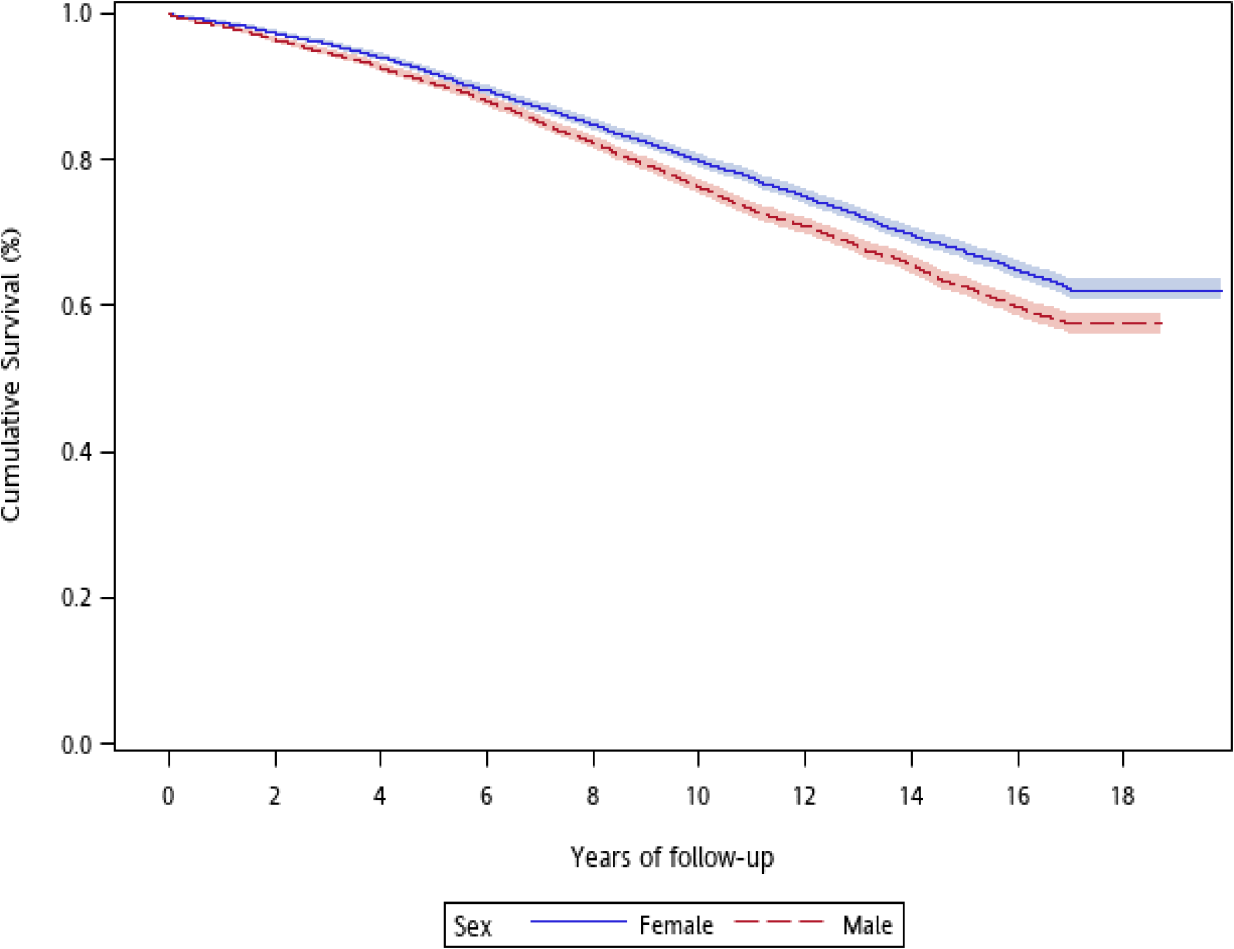
Sex-specific survival curves in patients with type 1 diabetes

**Figure 2.**
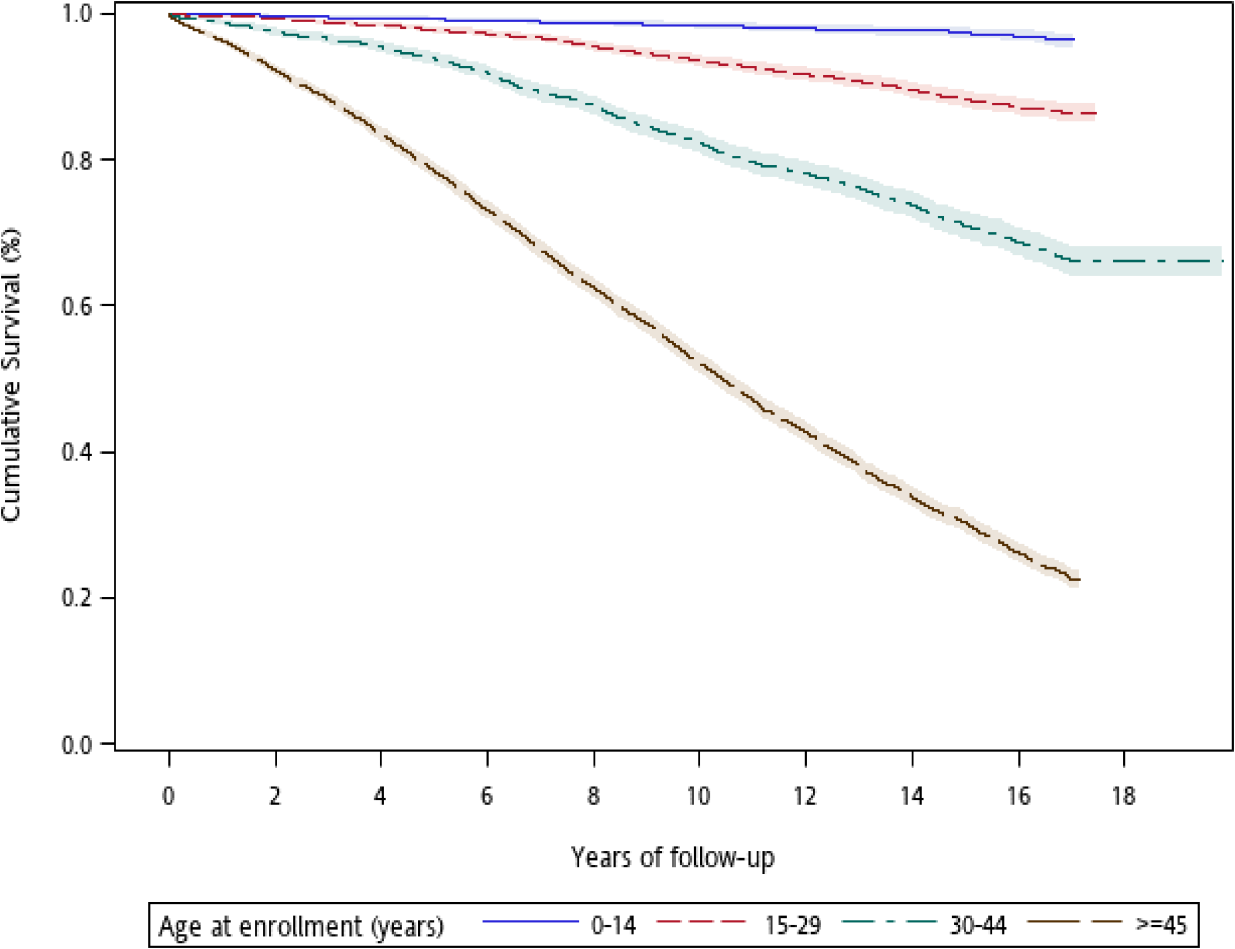
Age-specific survival curves in patients with type 1 diabetes

The study cohort included 7,696 prevalent (44.74%) and 9,507 incident (55.26%) cases of type 1 diabetes, with slight female dominance. The mean age at cohort enrollment was 33.05 (standard deviation [SD], 21.41; median, 28) years. In up to 17 years of follow-up, 4,916 patients died from all-cause at a mean age of 62.37 (SD, 16.68) years. Among the deceased individuals, 65 (1.32%) died at ages <20 years, and 2,556 (51.99%) died at 65 years or older (eTable 2). Cumulative survival risk were significantly different between males and females (*P* < 0.001, log-rank test) (Figure 1) and across ages at enrollment (*P* < 0.001, log-rank test) (Figure 2).

Diabetes was the leading UCOD (*n* = 1,482), which accounted for 30.15% of the total deaths, followed by cancer (*n* = 1,007, 20.48%), circulatory diseases (*n* = 646, 13.14%) and renal diseases (*n* = 563, 11.45%). The distributions of UCODs varied with age at death, in which the proportion of deaths from violence or accidents (including suicide) and diabetes were considerably higher in children (0–14 years) than in patients of older age groups. The proportions of deaths from circulatory diseases, renal diseases, infectious diseases, and pneumonia were higher among patients aged ≥45 years; the proportion of deaths from circulatory diseases increased with age (eFigure 1).

Distributions of UCOD were essentially similar between male and female subjects, with a slightly greater proportion of deaths from cancer noted in males and diabetes noted in females (eFigure 2). The proportions of deaths from circulatory diseases and infection increased over the study period, but the proportions of diabetes and chronic liver disease decreased (eFigure 3).

The mortality rate of patients with type 1 diabetes was 26.93 per 1,000 person-years for the entire patient group, 29.25 per 1,000 person-years for males, and 24.90 per 1,000 person-years for females (Table 1). The UCOD with higher mortality rates in patients with type 1 diabetes were diabetes, cancer, and circulatory disease (8.12, 5.52, and 3.54 per 1,000 person-years, respectively). The leading causes of death with higher mortality rate were similar between males and females. Cancer in liver and intrahepatic bile ducts was the most frequent UCOD among listed site-specific cancers in both males and females.

**Table 1.** All-cause and cause-specific standardized mortality ratio in patients with type 1 diabetes

	Total (*n* = 17,203)					Female (*n* = 9,008)					Male (*n* = 8,195)				
		
	Obs.	Mortality rate (10^3^ person-years)	Age and sex standardized mortality ratio			Obs.	Mortality rate (10^3^ person-years)	Age standardized mortality ratio			Obs.	Mortality rate (10^3^ person-years)	Age standardized mortality ratio		
		
			**Estimate**	95% CI				**Estimate**	95% CI				**Estimate**	95% CI	
All-cause mortality	4,916	26.93	**4.16**	4.04,	4.28	2,405	24.90	**4.62**	4.44	4.81	2,511	29.25	**3.79**	3.65	3.94
Circulatory diseases^a^	646	3.54	**2.52**	2.33,	2.72	341	3.53	**2.83**	2.54	3.15	305	3.55	**2.24**	2.00	2.51
Heart disease	286	1.57	**2.19**	1.94,	2.45	149	1.54	**2.45**	2.07	2.88	137	1.60	**1.95**	1.64	2.31
Cerebrovascular disease	304	1.67	**2.84**	2.53,	3.18	158	1.64	**3.11**	2.65	3.64	146	1.70	**2.60**	2.20	3.06
Hypertension without heart disease	44	0.24	**4.07**	2.96,	5.47	26	0.27	**4.58**	2.99	6.70	18	0.21	**3.52**	2.08	5.56
Other circulatory diseases	12	0.07	**1.52**	0.79,	2.66	8	0.08	**2.49**	1.07	4.90	4	0.05	**0.86**	0.23	2.20
Malignant neoplasm^a^	1,007	5.52	**2.94**	2.76,	3.13	409	4.23	**2.87**	2.60	3.16	598	6.97	**2.99**	2.76	3.24
Stomach	36	0.20	**1.73**	1.21,	2.39	22	0.23	**2.68**	1.68	4.06	14	0.16	**1.11**	0.61	1.86
Pancreas	59	0.32	**4.95**	3.76,	6.38	22	0.23	**3.87**	2.43	5.86	37	0.43	**5.92**	4.17	8.17
Liver and intrahepatic bile ducts	231	1.27	**3.46**	3.03,	3.94	79	0.82	**3.51**	2.78	4.37	152	1.77	**3.44**	2.91	4.03
Bronchus and lung	61	0.33	**2.30**	1.76,	2.96	22	0.23	**2.25**	1.41	3.41	39	0.45	**2.33**	1.66	3.19
Colon, rectum, and anus	111	0.61	**3.59**	2.96,	4.33	53	0.55	**3.60**	2.69	4.70	58	0.68	**3.59**	2.73	4.64
Prostate (males only)	29	0.16	**4.35**	2.92,	6.25	—	—	—	—	—	29	0.34	**4.35**	2.92	6.25
Ovary (females only)	6	0.03	**3.87**	1.41,	8.43	6	0.06	**3.87**	1.41	8.43	—	—	—	—	—
Bladder	21	0.12	**3.41**	2.11,	5.21	4	0.04	**1.64**	0.44	4.20	17	0.20	**4.58**	2.66	7.33
Breast (females only)	47	0.26	**3.59**	2.64,	4.77	47	0.49	**3.59**	2.64	4.77	—	—	—	—	—
Kidney	8	0.04	**1.47**	0.63,	2.89	2	0.02	**0.74**	0.08	2.68	6	0.07	**2.17**	0.79	4.73
Cervix uteri (females only)	28	0.15	**3.57**	2.37,	5.16	28	0.29	**3.57**	2.37	5.16	—	—	—	—	—
Other sites	370	2.03	**2.55**	2.30,	2.83	124	1.28	**2.30**	1.91	2.74	246	2.87	**2.71**	2.38	3.07
Diabetes Mellitus^*^	1,482	8.12	**16.45**	15.63,	17.31	786	8.14	**14.83**	13.81	15.90	696	8.11	**18.78**	17.41	20.23
Renal diseases	563	3.08	**14.48**	13.31,	15.73	303	3.14	**14.06**	12.52	15.74	260	3.03	**15.01**	13.24	16.95
Violence and accidents	85	0.47	**1.35**	1.08,	1.67	36	0.37	**1.83**	1.28	2.53	49	0.57	**1.14**	0.84	1.50
Suicide	38	0.21	**1.30**	0.92,	1.78	14	0.14	**1.34**	0.73	2.25	24	0.28	**1.27**	0.81	1.89
Infection	128	0.70	**4.59**	3.83,	5.46	71	0.74	**6.08**	4.75	7.67	57	0.66	**3.52**	2.67	4.56
Pneumonia	145	0.79	**3.00**	2.53,	3.53	62	0.64	**3.13**	2.40	4.02	83	0.97	**2.90**	2.31	3.60
Chronic hepatitis and liver cirrhosis	220	1.21	**4.91**	4.29,	5.61	80	0.83	**5.10**	4.05	6.35	140	1.63	**4.81**	4.05	5.68
Chronic obstructive pulmonary disease	63	0.35	**1.51**	1.16,	1.93	31	0.32	**2.26**	1.54	3.21	32	0.37	**1.14**	0.78	1.61
Any other cause	539	2.95	**2.70**	2.48,	2.94	272	2.82	**2.97**	2.63	3.35	267	3.11	**2.47**	2.18	2.79

CI, confidence interval; Obs, observed number; pys, person-years.

^a^Only selected diseases under this category were listed in sub-items.

^*^There were 861 records coded by ICD9-CM and 470 records coded by ICD10 (a total of 1,331 records [89.8%]) without specific classifications according to the 4^th^ digit in ICD codes.

Compared with the general population, patients with type 1 diabetes were at a significantly increased risk of all-cause mortality (SMR 4.16; 95% CI, 4.04–4.28). Cause-specific analysis indicated that the most increased SMR was observed for diabetes (SMR, 16.45), followed by renal disease (SMR, 14.48), chronic hepatitis and liver cirrhosis (SMR, 4.91), infection (SMR, 4.59), and pneumonia (SMR, 3.00). Cancer and circulatory diseases also exhibited an increased SMR (SMR, 2.94 and 2.52, respectively). Significantly increased SMRs were observed for the specific circulatory diseases, including heart disease, cerebrovascular disease, and hypertension without heart disease, as well as for listed site-specific cancers except kidney neoplasm. Among the cancer site-specific SMRs, SMR for pancreas was mostly increased at 4.95. Type 1 diabetes was also associated with a significantly increased SMR for violence and accidents (SMR, 1.35) and COPD (SMR, 1.51) (Table 1).

Sex-specific SMRs are also shown in Table 1. Male and female type 1 diabetes were significantly associated with elevated all-cause mortality with SMR of 3.79 and 4.62, respectively. Both male and female patients had significantly increased SMRs for diabetes, renal diseases, and cancer. Although male and female genders were associated with significantly increased SMRs of circulatory diseases, infection, pneumonia, and chronic hepatitis and liver cirrhosis, female patients had greater SMRs of the above causes than males.

The absolute mortality rate and all-cause SMRs were also significantly increased for all ages at enrollment (Table 2). The most increased SMR was observed for patients aged 15–29 years (SMR, 8.46), followed by ages of 30–44 years (SMR, 8.08) and 0–14 years (SMR, 5.37); SMR was the least for those enrolled at ≥45 years (SMR, 3.57). Significantly increased SMRs were noted for circulatory disease, cancer, diabetes, and pneumonia in all age groups. A substantial increase in SMR of renal diseases was observed among patients aged 15–29 years (SMR, 98.86), 30–44 years (SMR, 48.73), and ≥45 years (SMR, 12.50). SMRs of infection and chronic hepatitis and liver cirrhosis were also significantly increased in the three order age groups and were similarly decreased among patients aged ≥45 years. SMR of diabetes was highest in the youngest group aged 0–14 years and decreasing with age. SMR of violence and accidents was significantly increased only for ages 15–29 years (SMR, 1.68), and SMR of suicide significantly increased only for ages 30–44 years (SMR, 2.35). SMR of COPD was only significantly elevated for ages ≥45 years (Table 2).

**Table 2.** All-cause and cause-specific sex-standardized mortality ratio in patients with type 1 diabetes according to age at enrollment

	0–14 years					15–29 years					30–44 years					≥45 years				

	Obs	MR	**SMR**	95% CI		Obs	MR	**SMR**	95% CI		Obs	MR	**SMR**	95% CI		Obs	MR	**SMR**	95% CI	
All-cause mortality	78	18.23	**5.37**	4.25,	6.70	427	91.95	**8.46**	7.42,	9.60	755	258.56	**8.08**	7.57,	8.61	3,656	681.96	**3.57**	3.46,	3.69

Circulatory disease	4	0.94	**5.15**	1.39,	13.19	36	7.75	**8.69**	6.08,	12.02	98	33.56	**7.21**	5.85,	8.78	508	94.76	**2.13**	1.95,	2.33
Malignant neoplasm	10	2.34	**5.66**	2.71,	10.41	26	5.60	**3.07**	2.00,	4.49	106	36.30	**3.15**	2.58,	3.81	865	161.35	**2.90**	2.71,	3.10
Diabetes	35	8.18	**397.91**	277.11,	553.41	186	40.05	**316.66**	272.78,	365.58	268	91.78	**84.54**	74.72,	95.29	993	185.23	**11.52**	10.81,	12.26
Renal diseases	0	0.00	**0**	NA		35	7.54	**98.86**	68.85,	137.49	64	21.92	**48.73**	37.53,	62.23	464	86.55	**12.50**	11.39,	13.69
Violence and accidents	8	1.87	**1.44**	0.62,	2.83	21	4.52	**1.68**	1.04,	2.57	16	5.48	**1.66**	0.95,	2.69	40	7.46	**1.14**	0.81,	1.55
Suicide	4	0.94	**3.10**	0.83,	7.93	7	1.51	**0.96**	0.38,	1.97	16	5.48	**2.35**	1.34,	3.81	11	2.05	**0.79**	0.39,	1.42
Infection	0	0.00	**0**	NA		8	1.72	**13.93**	6.00,	27.44	26	8.90	**17.07**	11.15,	25.02	94	17.53	**3.67**	2.97,	4.49
Pneumonia	2	0.47	**11.51**	1.29,	41.56	10	2.15	**18.86**	9.03,	34.69	18	6.16	**13.3**	7.88,	21.04	115	21.45	**2.48**	2.05,	2.98
Chronic hepatitis and liver cirrhosis	1	0.23	**15.88**	0.21,	88.34	12	2.58	**4.69**	2.42,	8.19	55	18.84	**6.18**	4.65,	8.04	152	28.35	**4.57**	3.88,	5.36
Chronic obstructive pulmonary disease	0	0.00	**0**	NA		1	0.22	**4.17**	0.05,	23.21	3	1.03	**4.49**	0.90,	13.13	59	11.01	**1.45**	1.10,	1.87
Any other cause	14	3.27	**3.12**	1.71,	5.24	85	18.30	**7.88**	6.30,	9.74	85	29.11	**5.25**	4.19,	6.49	355	66.22	**2.11**	1.90,	2.34

CI, confidence interval; MR, mortality rate (per 1,000 person-years); NA, not applicable due to limited number of deaths; Obs, observed number; SMR, Standardized Mortality Ratio.

## DISCUSSION

### Main findings

Cancer accounted for 20.48% of total deaths, followed by circulatory diseases (13.14%) and renal diseases (11.45%) in our sample. The all-cause SMR was significantly elevated at 4.16, with a greater all-cause SMR noted in females than in males (4.62 vs 3.79). Diabetes and renal diseases were associated with the most increased cause-specific SMR in both genders.

### Overall and cause-specific analysis

Apart from acute diabetes complications, cancer was the leading UCOD among type 1 diabetes in Taiwan, which is dissimilar to the findings of previous studies that CVD remains common and leads to premature mortality in type 1 diabetes.^1^^,^^12^^,^^23^ In 27 years after entry into the Diabetes Control and Complications Trial (DCCT) trial, 107 deaths were noted; the most common causes of death were CVD (22%), cancer (20%), and acute complications (18%).^12^ Livingstone et al^23^ also found that ischemic heart disease (IHD) was most related to the estimated loss in life expectancy among type 1 diabetes (36% in men, 31% in women). However, deaths from cancer also appear high in the above-mentioned two studies.^24^ Dissimilarity in distributions of UCODs among type 1 diabetes reported by different studies is likely due to different baseline disease incidence rates across different populations.

The mortality rate (26.4 per 1,000 person-years) reported in our study was high. Most of previous studies recruited young incident cases of type 1 diabetes at baseline and followed the participants to their thirties, the studies reported mortality rates ranged from 0.7 to 6.75 per 1,000 person-years.^12^^,^^23^^,^^25^^–^^28^ Our study observed prevalent cases, with mean age of 33 years-old at enrollment, and observed for 11 years on average. The older age and longer disease duration in our study subjects may substantially contribute to the high mortality rate.

Despite the potential methodological problems with comparing SMRs,^29^ disparity in all-cause SMR reported in different countries and population is apparent. In the United States, mortality in the DCCT conventional therapy group was significantly greater than that in the general population (SMR 1.31; 95% CI, 1.05–1.65).^30^ A greater all-cause SMR was observed in studies conducted in Northern Ireland (SMR 2.96; 95% CI, 2.29–3.82),^31^ Brazil (SMR 3.13; 95% CI, 2.35–4.08),^32^ and Wales (SMR 2.91; 95% CI, 1.96–4.15).^25^ The all-cause SMR noted in our study (SMR, 4.16) was comparable to the SMRs estimated from a Danish study (SMR 4.8; 95% CI, 3.5–6.2)^26^ and a study conducted in Yorkshire, United Kingdom (SMR 4.3; 95% CI, 3.8–4.9).^13^ Disparity in all-cause SMR also existed within a country over time.^5^^,^^6^^,^^27^

Our study showed a very high SMR (14.48) for renal disease. Diabetes mellitus is considered the most common cause of end-stage renal disease (ESRD). People with diabetes and diabetic nephropathy are more vulnerable to multiple medical conditions than those without diabetes at similar stages of chronic kidney disease. People with diabetes and chronic kidney disease are also prone to hospitalization with infections and acute kidney injury.^33^ Onda et al^1^ found that 36.3% of 113 deceased individuals with type 1 diabetes and ESRD had ESRD as the leading cause of death. Taiwan is among the countries with high incidence (range of 407–476 per 10^6^) and prevalence rates (2,525–3,317 per 10^6^) of ESRD in the world between 2008 and 2015.^34^ A disproportionally high prevalence of ESRD in people living with diabetes in Taiwan and a high association between diabetes and chronic kidney disease mortality might have contributed to the high SMR for renal diseases noted in our study.

### Sex-specific analysis

Our study noted a higher all-cause SMR in female patients than in male ones (4.62 vs 3.79). Compared with males, female patients had a higher cause-specific SMR for circulatory disease (2.83 vs 2.24) and infection (6.08 vs 3.52) but had comparable SMRs for cancer (2.87 vs 2.99) and renal disease (14.06 vs 15.01). Two Norwegian studies found the SMR for all-causes were similar in both genders.^6^^,^^27^ In addition, slight sex difference in all-cause SMR was observed in a United Kingdom study that reported SMRs of 4.4 (95% CI, 3.8–5.2) and 4.0 (95% CI, 3.2–5.2) for males and females, respectively.^13^

Despite the aforementioned inconsistency, Lung et al^9^ conducted meta-analyses based on 26 studies and found that RRs of all deaths were 3.25 (95% CI, 2.82–3.73) and 4.54 (95% CI, 3.79–5.45) for men and women, respectively. A recent study conducted in Northern Ireland by Morgan et al^31^ also found that women had a significantly higher excess risk of mortality than men with SMRs of 5.35 (95% CI, 3.61–7.64) and 2.03 (95% CI, 1.36–2.91), respectively. Harjutsalo et al^35^ examined long-term mortality from IHD in a Finnish population-based cohort with type 1 diabetes. Females had significantly greater SMR than males, and a difference in SMR between sexes was striking in the early-onset cohort (women: 52.8, 95% CI, 36.3–74.5; men: 12.1, 95% CI, 9.2–15.8).^35^

A high SMRs of all-cause and circulatory causes could be due to the “*catching-up*” effect in women after having diabetes, despite women usually have a far lower risk of all-cause and CVD mortality than men for much of their lifespan. A meta-analysis of 214,114 type 1 diabetes reported that the pooled women-to-men ratio of the SMR was 1.37 (95% CI, 1.21–1.56) for all-cause mortality, 1.44 (95% CI, 1.02–2.05) for renal disease, 1.86 (95% CI, 1.62–2.15) for CVDs, and even more extreme for coronary heart disease (2.54, 95% CI, 1.80–3.60).^36^

### Age-specific analysis

A Norwegian study by Gagnum et al^6^ observed a cohort with type 1 diabetes and found that 249 (3.2%) died during a mean follow-up of 16.8 years. The SMR for all-causes was 3.6 (95% CI, 3.1–4.0), which increased by attained age.^6^ However, such findings were inconsistent with our findings that showed high SMR in ages 15–29 years.

Most previous studies found that the leading cause of death before the age of 30 years was acute complications, while CVD was predominant after the age of 30 years. However, deaths attributable to acute complications, such as infection, were still important in all age groups.^5^

Harjutsalo et al^35^ found that the RR of dying from IHD was greatest in patients aged <40 years and 40–60 years in the early- and late-onset cohorts, respectively. We used age at type 1 diabetes registration in CID rather than age at disease onset. Thus, the different age-specific SMRs cannot be compared straightforward since the risk of IHD in older prevalent cases was expected to be higher. However, our study showed significantly elevated SMR for suicide among patients aged 30–44 years and significantly elevated SMR for violence and accidents among patients aged 15–29 years. Associations between type 1 diabetes and psychiatric disorders among younger patients have been well documented.

### Strengths and limitations

This population-based study is the first to investigate the leading causes of death in Asian populations who have relatively low incidence of type 1 diabetes. The use of national medical claim data and death registry ensures the representativeness of type 1 diabetes population and complete ascertainment of deceased individuals. A sufficiently large type 1 diabetes population size also facilitates age and sex specific analyses without compromising statistical power.

However, our study sample was mixed with incident and prevalent cases of type 1 diabetes, which mixed the concept of incidence and survival and resulted in difficulty comparing our results with previous studies that mostly started the follow-up from the date of type 1 diabetes diagnosis. The other limitation was lack of complete adjustment for potential prognostic factors after type 1 diabetes, such as treatment regimens and lifestyles, which also limited the interpretation of our study results. Finally, a substantial records in our study indicated that diabetes was the UCOD of the deceased with type 1 diabetes without further information, the vague attribution might bias the statistics about UCOD. However, we believed that the UCOD of these records might be mostly related to acute complications of diabetes which were rarely coded as UCOD and should be largely underestimated in our study.

Over 17 years of follow-up, patients with type 1 diabetes in Taiwan experienced a significantly elevated mortality from all-cause and various UCODs. Cancer accounted the largest number of total deaths, while renal disease was associated with the greatest and substantially elevated SMR. Apart from traditionally recognized causes of death associated with type 1 diabetes, such as CVDs and cancer, certain UCODs, such as chronic hepatitis or liver cirrhosis and COPD in older patients, as well as accidents and suicide in younger patients, were also significantly associated with type 1 diabetes. Clinicians should consider the specific UCOD in delivering treatment and health care to patients with type 1 diabetes.

## APPENDIX A. SUPPLEMENTARY DATA

eTable 1. ICD codes for diseases analyzed in this study

eTable 2. Characteristics of study subjects

eFigure 1. Age-specific proportions of major causes of death among patients with type 1 diabetes in the period of 1998–2014.

eFigure 2. Sex-specific proportions of major causes of death among patients with type 1 diabetes in the period of 1998–2014.

eFigure 3. Secular trend in proportions of major causes of death among patients with type 1 diabetes in the period of 1998–2014.
