# Supplementary material for: Overall and Cause-Specific Mortality in Patients With Type 1 Diabetes Mellitus: A Population-Based Cohort Study in Taiwan From 1998 Through 2014
Source: J Epidemiol. 2021 Sep 5;31(9):503–10. doi: 10.2188/jea.JE20200026 (PMC8328860; doi:10.2188/jea.JE20200026)
Supplement: Supplementary file 1 [file je-31-503-s001.pdf]

eTable 1. ICD codes for diseases analyzed in this study

Diseases	ICD-9-CM	ICD-10-CM
All-cause mortality		
Circulatory diseases	390–459	I00–I99
Heart disease	390–398, 402–404, 410–429	I00–I09, I11–I13, I20–I51
Cerebrovascular disease	430–438	I60–I69
Hypertension without heart disease	401, 403	I10, I12
Malignant neoplasm	140–239	C00–C97
Stomach	151	C16
Pancreas	157	C25
Liver and intrahepatic bile ducts	155.0–155.2	C220–C224, C227, C229
Bronchus and lung	162.2–162.5, 162.8 –162.9	C34
Colon, rectum, and anus	153, 154.0–154.3, 154.8–154.9	C18–C21, C260
Prostate (males only)	185	C61
Ovary (females only)	183.0	C56
Bladder	188	C67
Breast (females only)	174	C50
Kidney	189	C64–C65
Cervix uteri (females only)	180	C53
Diabetes	250	E10, E11, E14
Renal diseases	580–589	N00–N07, N17–N19, N25–N27
Violence and accidents	800–949	V00–V99, X00–X59, X85–X99, Y00–Y09, Y85–Y86, Y871
Suicide	950–959	X60–X84, Y870
Infectious disease	001–139	A00–A41
Pneumonia, organism unspecified	486	J18
Chronic hepatitis or liver cirrhosis	571	K70, K73–K74
COPD	491–496	J40–J47

COPD, Chronic Obstructive Pulmonary Disease; ICD-9-CM, International Classification of Diseases, Ninth Revision Clinical Modification; ICD-10-CM, International Classification of Diseases, Tenth Revision Clinical Modification.

eTable 2. Characteristics of study subjects

	n	%
Total	17,203	100.00
Incident cases between 1998 and 2014		
No[a]	7,696	44.74
Yes	9,507	55.26
Calendar year of enrollment[b]		
Prior to 2003	11,080	64.41
2003–2006	2,550	14.82
2007–2010	2,033	11.82
2011–2014	1,540	8.95
Sex		
Female	9,008	52.36
Male	8,195	47.64
Age at cohort enrollment, years		
0–14	4,278	24.87
15–29	4,644	27.00
30–44	2,920	16.97
>=45	5,361	31.16
Mean (SD)	33.05 (21.41)	
Median (Q1–Q3)	28 (15–50)	
Years of follow-up		
Mean (SD)	10.61 (5.34)	
Median (Q1–Q3)	11.08 (6.07–16.19)	
Survival status by the end of 2014		
Survivors	12,287	71.42
Non-survivors	4,916	28.58
Age at death, years		
0–19	65	1.32
20–44	716	14.56
45–64	1,579	32.12
65+	2,556	51.99
Mean (SD)	62.37 (16.68)	
Median (Q1–Q3)	65 (52–75)	

SD, standard deviation.

[a] Prevalent cases of type 1 diabetes diagnosed prior to 1998.

[b] Date of first-time inpatient/outpatient appearance with a type 1 diabetes diagnosis in the period of 1998–2014.

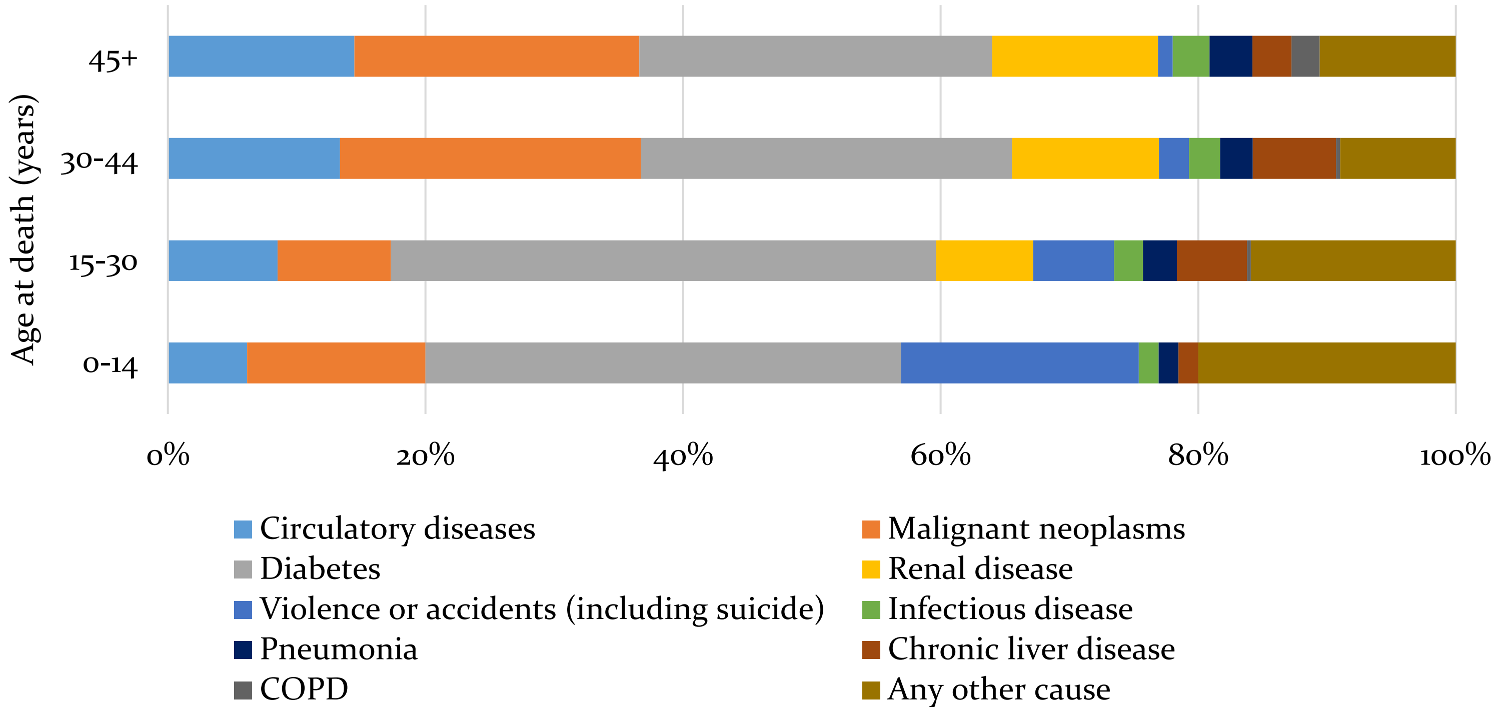

eFigure 1. Age-specific proportions of major causes of death among patients with type 1 diabetes in the period of 1998–2014.

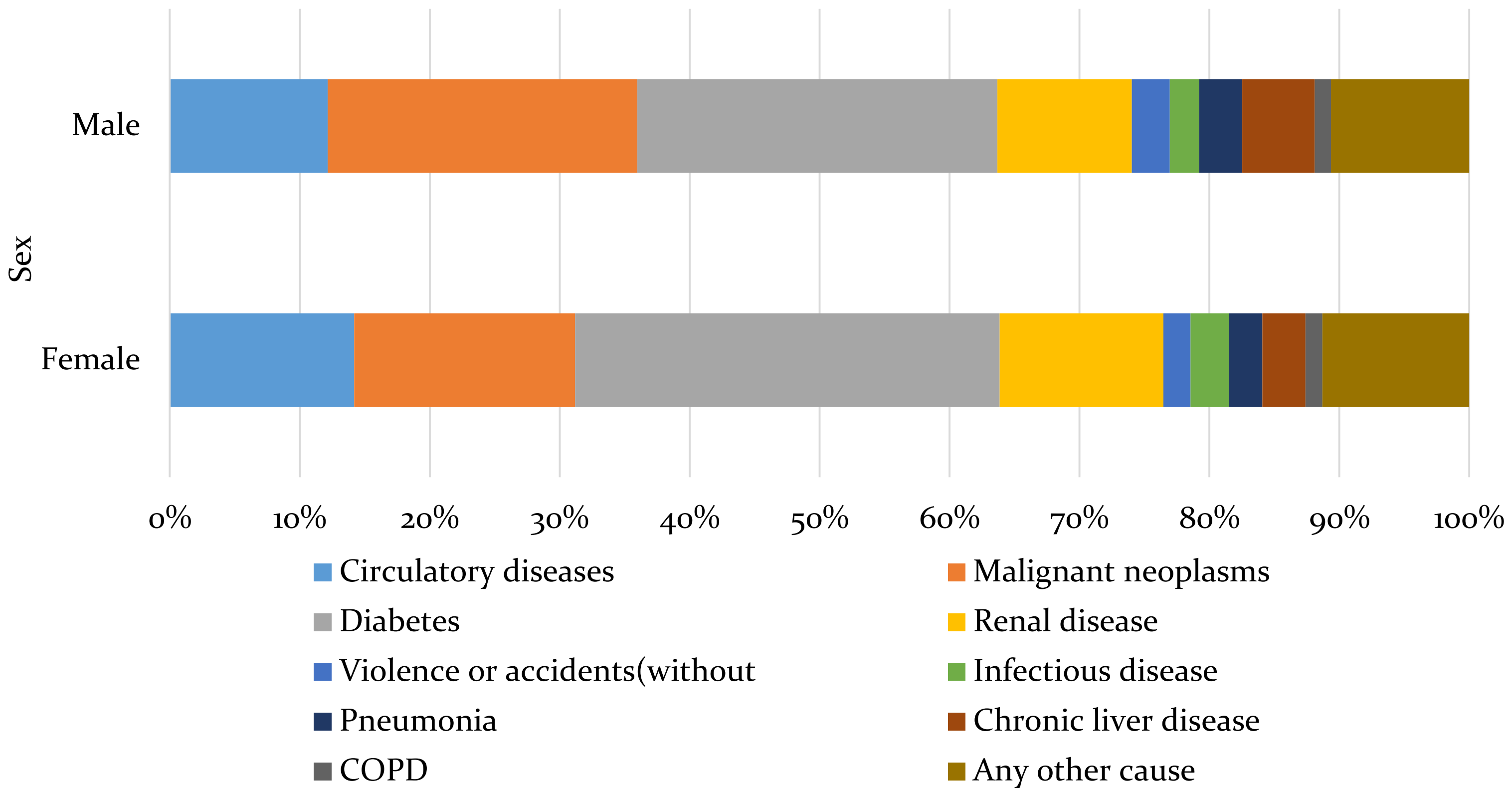

eFigure 2. Sex-specific proportions of major causes of death among patients with type 1 diabetes in the period of 1998–2014.

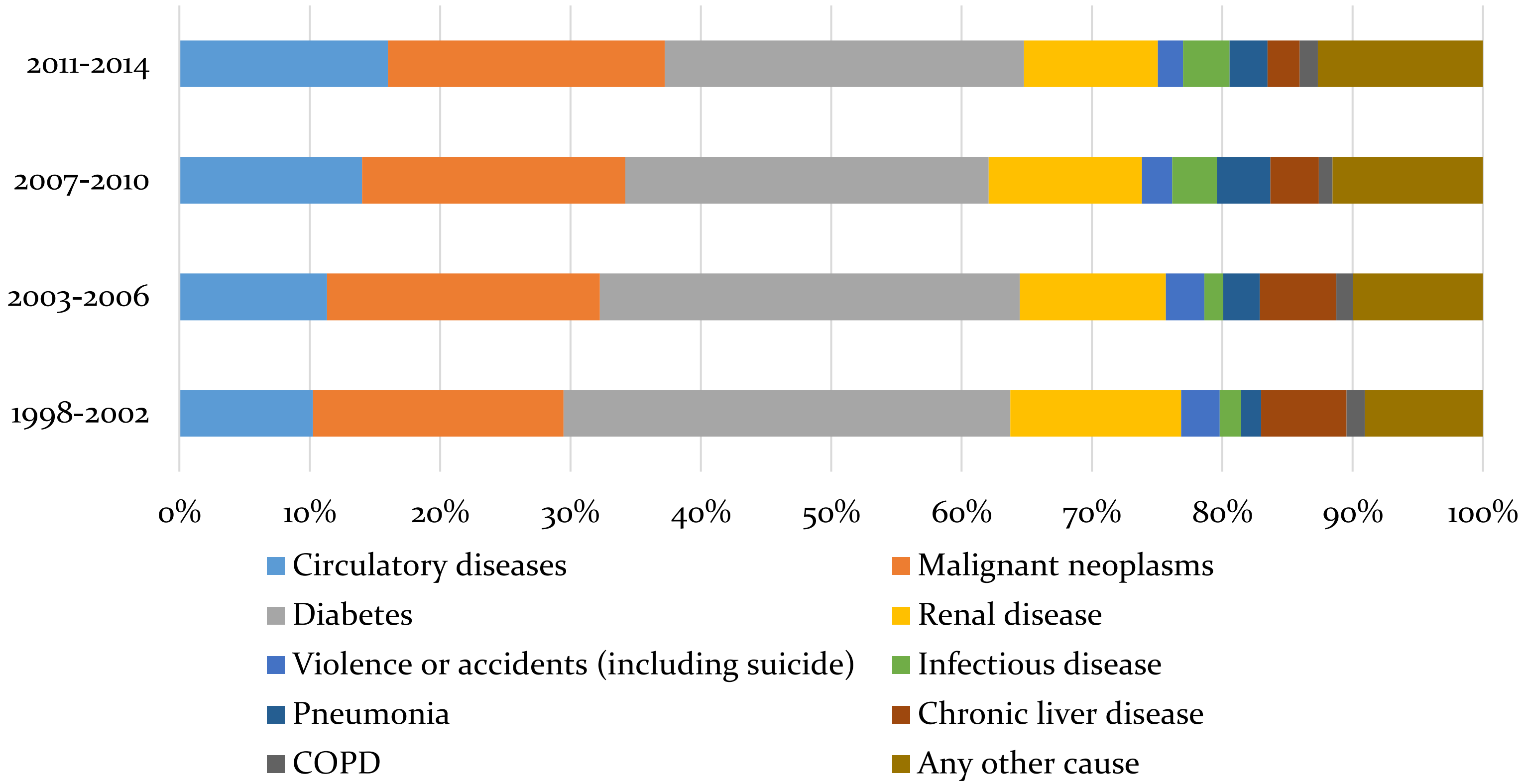

eFigure 3. Secular trend in proportions of major causes of death among patients with type 1 diabetes in the period of 1998–2014.
